# Orbitofrontal ^**18**^F-DOPA Uptake and Movement Preparation in Parkinson's Disease

**DOI:** 10.1155/2015/180940

**Published:** 2015-06-11

**Authors:** Lucio Marinelli, Arnoldo Piccardo, Laura Mori, Silvia Morbelli, Nicola Girtler, Antonio Castaldi, Agnese Picco, Carlo Trompetto, Maria Felice Ghilardi, Giovanni Abbruzzese, Flavio Nobili

**Affiliations:** ^1^Department of Neuroscience, Rehabilitation, Ophthalmology, Genetics, Maternal and Child Health, University of Genoa, Largo Daneo 3, 16132 Genoa, Italy; ^2^Nuclear Medicine Unit, Galliera Hospital, Mura delle Cappuccine 14, 16128 Genoa, Italy; ^3^Nuclear Medicine Unit, Department of Health Sciences, IRCCS AOU San Martino, IST, University of Genoa, Largo Rosanna Benzi 10, 16132 Genoa, Italy; ^4^Clinical Psychology and Psychotherapy Unit, IRCCS AOU San Martino, IST, Largo Rosanna Benzi 10, 16132 Genoa, Italy; ^5^Department of Diagnostic and Interventional Neuroradiology, Galliera Hospital, Mura delle Cappuccine 14, 16128 Genoa, Italy; ^6^Department of Physiology, Pharmacology and Neuroscience, City University of New York Medical School, 160 Convent Avenue, New York, NY 10031, USA

## Abstract

In Parkinson's disease (PD) degeneration of mesocortical dopaminergic projections may determine cognitive and behavioral symptoms. Choice reaction time task is related to attention, working memory, and goal-directed behavior. Such paradigm involves frontal cortical circuits receiving mesocortical dopamine which are affected early in PD. The aim of this study is to characterize the role of dopamine on the cognitive processes that precede movement in a reaction time paradigm in PD. We enrolled 16 newly diagnosed and untreated patients with PD without cognitive impairment or depression and 10 control subjects with essential tremor. They performed multiple-choice reaction time task with the right upper limb and brain ^18^F-DOPA PET/CT scan. A significant inverse correlation was highlighted between average reaction time and ^18^F-DOPA uptake in the left lateral orbitofrontal cortex. No correlations were found between reaction time and PD disease severity or between reaction time and ^18^F-DOPA uptake in controls. Our study shows that in PD, but not in controls, reaction time is inversely related to the levels of dopamine in the left lateral orbitofrontal cortex. This novel finding underlines the role of dopamine in the lateral orbitofrontal cortex in the early stages of PD, supporting a relation between the compensatory cortical dopamine and movement preparation.

## 1. Introduction

Early manifestations of Parkinson's disease (PD) may include cognitive and affective abnormalities involving multiple domains such as attention, working memory, visuospatial abilities [1], apathy and depression [2]. Most of these clinical features have been related to a frontal dysexecutive syndrome whose neurobiological substrate can be referred to as an unbalanced mesocortical dopaminergic activity [3].

The neurodegenerative process in PD involves nigrostriatal dopaminergic neurons but also those originating from the ventral-tegmental area and projecting to the frontal cortex through the mesocortical pathway [4]. While the nigrostriatal network is mainly responsible for the motor aspects of PD, the mesocortical network is essentially involved in cognitive and behavioral symptoms [5]. Both in human and animal models, the role of dopamine in motivation, reward, impulsivity, attention, working memory, and goal-directed behavior has been highlighted [6–10]. The orbitofrontal cortex is involved in goal-directed behavior and has a pivotal role within the mesocortical pathway. In fact, orbitofrontal cortex receives dopaminergic input from the ventral-tegmental area and modulates the dopaminergic output of the mesocortical pathway [9]. Choice reaction time tasks involve frontal cortical circuits controlling attention, working memory, and goal-directed behavior; these resources, which are also related to learning abilities, seem to be decreased in PD [11]. The aim of this study is to characterize the role of dopamine (as measured by ^18^F-DOPA PET) during motor preparation, investigated using a multiple-choice reaction time paradigm in early untreated PD patients.

## 2. Methods

### 2.1. Subjects

Subjects were sixteen patients affected by idiopathic PD consistent with established guidelines (age 67 ± 7, mean ± SD), 3 females, H&Y stages 1-2, and UPDRS-III 16 ± 5. They were all right-handed (as assessed by the Edinburgh Handedness Inventory), newly diagnosed, and drug naïve. All of them showed asymmetric presentation with the left side more affected in 11 patients, the right side in the remaining 5. Ten patients with a clinical diagnosis of essential tremor were enrolled as controls (age 68 ± 10, 6 females). None had significant cognitive impairment, as confirmed by a neuropsychological assessment including the Mini-Mental State Examination (MMSE) where all patients scored more than 26 (Table 1). The presence of significant depressive symptoms was also excluded by the 15-item Geriatric Depression Scale (GDS) where all patients scored less than 6.

Although healthy subjects would have been more suitable as control subjects, it is known that patients with essential tremor have a normal ^18^F-DOPA PET [12]. It must be said that there are heavy ethical and regulatory difficulties in studying healthy controls with radioactive compounds. Moreover, in the clinical setting the differential diagnosis is often between PD and essential tremor, rather than healthy subjects, and thus essential tremor represents the usual group PD patients are compared with.

The present study has been carried out in accordance with the Code of Ethics of the World Medical Association (Declaration of Helsinki) for experiments involving humans; a written informed consent was obtained from all participants. The study was approved by the local Ethics Committee.

### 2.2. Motor Task

**Figure 1:**
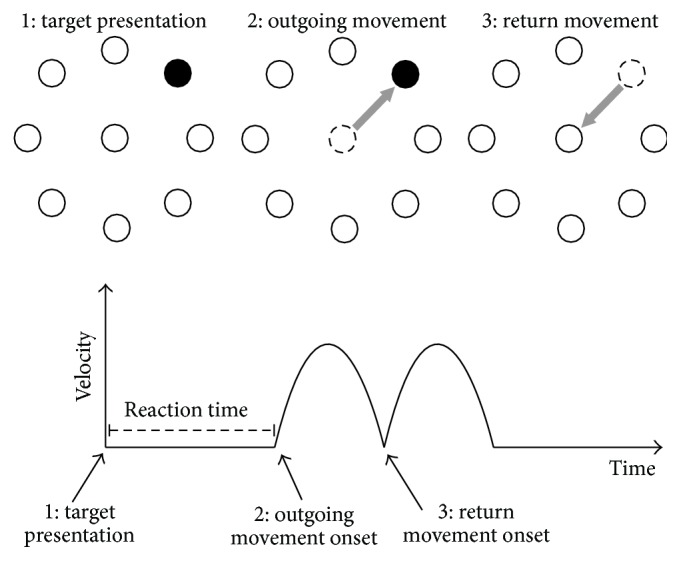
Motor task. (1) One of eight targets turns black (target presentation); then (2) the patient moves the cursor toward the target as soon as possible (outgoing movement) and (3) returns to the centre right after reaching the target (return movement). The reaction time is the time required to start the movement after target presentation.

All subjects were naïve to the task and therefore performed a short training session, leading to a smooth performance. It must be underlined that the training phase was short enough so that the subjects still needed to exert significant attentional effort and did not reach an automatic habitual control during the following motor task. The experimental setup has been described in detail in previous works [13]; briefly, subjects moved a cursor on a digitizing tablet with their right (dominant) hand. Movements were out and back from a central starting point to one of eight radial targets displayed on a computer screen. The targets' distance from the central point was 4.8 cm. One of eight circular empty targets turned black on the screen in synchrony with a tone at fixed intervals of 1.5 s in unpredictable order. Subjects were asked to reach for the target making out and back movements “as soon as possible” and “as fast as possible,” thus minimizing reaction time and movement duration. Instructions were also to make the out and back movements without corrections, reversing sharply inside each target circle. When the subjects reached the target within 1.5 s, before the next one turning black, the target circle turned gray, indicating a successful hit. Subjects received a feedback about their performance at the end of each block. The described experimental paradigm can be considered a multiple-choice reaction time task since the subjects deploy attention toward eight targets and then reach for the one that turns black. All subjects performed 160 consecutive reaching movements into two blocks of 80 movements each. We measured reaction time during reaching movements as the time between the target appearance and the movement onset; for each subject we computed the average reaction time across the 160 movements (Figure 1).

The Motor Task Manager software (ETT, Electronic Technology Team, Genoa, Italy) controlled the protocol execution, path acquisition, and offline analysis of the data.

### 2.3. Image Protocol

#### 2.3.1. ^18^F-DOPA PET/CT

The patients and control subjects fasted for at least 4 hours before the start of ^18^F-DOPA PET/CT acquisition. On arrival subjects were given oral carbidopa (2.5 mg/kg), and one hour later, they received 185 MBq of ^18^F-DOPA intravenously. ^18^F-DOPA (IASOdopa, IASON Labormedizin Gesmbh & Co.Kg, Graz-Seiersberg, Austria) was produced as previously described [14]. Brain scans were acquired 90 minutes after injection [15]. Data were obtained using a dedicated PET/CT system (Discovery STE, GE Medical Systems, Milwaukee, WI, USA) using the 3D mode with a scanning time of 30 minutes. A nondiagnostic, low-dose CT scan (120 kV, 80 mA, 0.6 s per rotation) was used for attenuation correction.

Static (single frame) acquisition was preferred to dynamic one because it is more practical in a clinical setting. As a matter of fact, ^18^F-DOPA brain kinetics is exceedingly complex. Different modeling approaches have been proposed and still research and discussion are open on these issues [16]. Meanwhile, static acquisition with normalization of uptake values on a reference region (cerebellum or occipital lobe) has been validated as a simpler and suitable approach for both research and clinical purposes [15, 17] and has been increasingly used in studies of Parkinsonian patients [18, 19]. Moreover, normalized uptake values on reference regions tightly correlate with kinetic parameters [15] and have similar diagnostic accuracy in PD as kinetic parameters [15, 17, 18]. Also, in PD similar diagnostic accuracy for occipital-normalized ^18^F-DOPA uptake as ^123^I-FP-CIT SPECT has been demonstrated [19–21]. The PET scan was performed within one month from the motor task.

#### 2.3.2. MRI

All patients and control subjects underwent brain Magnetic Resonance Imaging (MRI) by means of a 1.5 T superconductive system (Signa HDxt, GE Healthcare, Milwaukee, WI) to acquire a sagittal T1-FFE sequence with the following parameters: TR 8.7 ms, TE 4.1 ms, flip angle 8°, FOV 256 mm, matrix 256 × 256, 150 sagittal slices (1.0-mm thick), and voxel size 0.98 × 0.98 × 1.6. White matter hyperintensities were graded according to the Wahlund's scale [22] and no patient scored more than 1 in each brain region.

### 2.4. Images Processing

All preprocessing and statistical analysis steps were performed using SPM8 package (Wellcome Department of Cognitive Neurology, London, UK) implemented in Matlab 6.5 (Mathworks, Natick, Massachusetts, USA).

Since it was shown that the use of a tracer-unmatched PET template to normalize brain scans may generate inconsistent results [23] and since SPM default brain PET template is H_2_^15^O template, a fully MRI-based normalization was applied [24]. Each ^18^F-DOPA PET scan was first coregistered to the pertinent MRI scan (six parameters, rigid body transformation) using the coregistration algorithm available in the SPM8 package. Each MR image was then spatially normalized to the SPM8 T1-MRI template using an affine plus nonlinear transformation, and the resulting deformation field was applied to the coregistered ^18^F-DOPA PET scan. The spatially normalized PET images were finally smoothed with a 6-mm isotropic Gaussian filter to blur individual anatomic variations and to increase the signal-to-noise ratio.

The voxel-based approach was chosen because it allows good spatial normalization of individual images to a stereotactic-normalized template, thus reducing the intersubject variability for anatomic topography, that is, allowing comparison of the same region among different subjects. This ability avoids systematic errors in ROI positioning or erroneous identification of striatal or extrastriatal regions in PET images with a low signal-to-noise ratio. Second, SPM tool interrogates all brain regions in an operator-independent way allowing estimating both striatal subregions and extrastriatal regions [25, 26].

### 2.5. Statistics

In order to fully explore correlation between dopaminergic (dys)function and average reaction time, both native images and images “flipped” with the more affected hemisphere (MAH) on the left side were independently submitted to the analysis. According to the clinical asymmetry in body impairment, since the right hemisphere was more affected in eleven PD patients and the left one in five, we explored also the hypothesis that possible correlation clusters derived from the relatively higher or lower involvement of the two hemispheres. Thus, after performing the first analyses we also repeated analyses after “flipping” the hemispheres so as to have the more affected hemisphere always on the right and the less affected one always on the left side of the brain. The MAH was defined as the contralateral one to the side of the body with prevalence of motor symptoms as assessed by the referring clinician. “Multiple Regression Analysis” was chosen between the possible designs available in “Basic Models” function of SPM8. This option allows the voxel-wise evaluation of the correlation between a variable of interest (average reaction time) and PET-assessed dopaminergic activity as well as nuisance effect (age, education, and UPDRS-III were included as nuisance variables to account for possible influence of these variables on both reaction time and PET data) both in PD and in control groups (UPDRS-III in PD only). Global calculation was performed on cerebellar counts previously obtained by means of MarsBaR, a region-of-interest analysis subtool of SPM [27]. SPM-T maps were displayed using an uncorrected *p* < 0.001 at peak level. This more liberal choice was adopted to avoid type II errors attributable to overconservative thresholds [28]. Given the exploratory nature of this analysis and considering the relatively low sensitivity of PET without repeated measures, higher thresholds could lead to false-negative results in PET studies. Clusters of correlations were regarded as significant if they survived at *p* < 0.05 threshold, FDR-corrected at cluster level. Only significant clusters containing at least 50 voxels were taken into consideration. Correction of SPM coordinates to match the Talairach coordinates was achieved by the subroutine implemented by Matthew Brett [29]. Brodmann areas (BA) were then identified at a range of 0 to 3 mm from the corrected Talairach coordinates of the SPM output isocenters, after importing the corrected coordinates, by Talairach client [30].

A factorial ANOVA was performed to compare reaction time between PD and controls, while the correlation between reaction time and UPDRS-III was computed with a linear regression analysis.

## 3. Results

All subjects successfully learned to perform the task, and their out and back movements were smooth with overlapping trajectories, returning to the center before the appearance of the next target. The average reaction time in the PD group was 302 ± 50 ms, while in the control group it was 341 ± 85 ms, with no significant difference between the two groups (*F*[1,24] = 2.2, *p* = 0.2). The reaction time was unrelated to the UPDRS-III scale.

**Figure 2:**
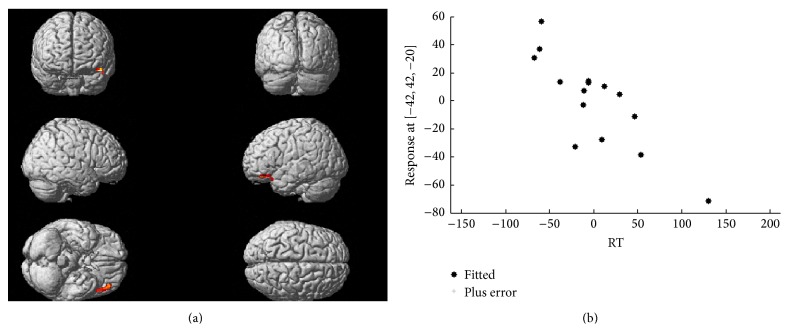
Correlation between ^18^F-DOPA uptake and reaction time. The upper panel shows whole brain voxel-wise correlation analysis depicting (superimposed to a brain rendering) the sites of significant inverse correlation between reaction time and PET-assessed dopaminergic activity. The cluster includes areas pertaining to the middle and inferior frontal as well as the superior temporal gyri in the left hemisphere. The lower panel shows the plot as automatically generated by SPM expressing the correlation between the two variables after transformation of the native values around 0.

DOPA uptake was significantly lower in PD than in control group in bilateral striatum, mainly in the more affected hemisphere. A significant inverse correlation was highlighted between average reaction time and PET-assessed dopaminergic activity in the left middle and inferior frontal gyri (BA 11 and 47), corresponding to the left lateral orbitofrontal cortex, and, to a lesser extent, in the left superior temporal gyrus (BA 38) as shown in Figure 2. The same correlation lost significance when images were flipped with the MAH on the left side. No significant correlation was found in any other brain regions. See Table 2 for further details and *Z* scores. In the control group, no correlation could be found between reaction time and PET-assessed dopaminergic activity in any brain area.

## 4. Discussion

This study demonstrates that the reaction time during a choice reaction time paradigm is inversely correlated to the dopaminergic activity in the left lateral orbitofrontal cortex in untreated patients in the early stages of PD but not in patients with essential tremor. Specifically, a higher reaction time corresponded to lower dopaminergic activity in the left lateral orbitofrontal cortex and vice versa. One of the most characteristic motor features of PD is the increased time in movement onset, as reflected experimentally with an increased reaction time especially in choice reaction time tasks [31]. Such slowness in movement preparation is less evident in the early stages of the disease, although slight between-subjects variability in reaction time may still be able to disclose early disruption of the brain network underlying movement preparation in PD [11]. Our PD patients were in the early stages of the disease and did not show a significant increase in reaction time if compared with those with essential tremor. This finding is not unexpected since reaction time can be increased also in essential tremor, where the altered functioning of brain areas such as the cerebellum could determine an increased reaction time involving neural mechanisms different from those of PD [32].

Reaction time encompasses the time required to detect and direct attention toward the highlighted target and to plan the appropriate response. Thus reaction time appears to be an ideal parameter to provide a temporal measure required by these cognitive processes. Since all the tested subjects were naïve to the task and underwent only a short preliminary training, their performance was still significantly linked to attentional resources. Recent studies show that attention is impaired in PD, particularly cognitive flexibility [33]. Attentional resources depend on the prefrontal dopaminergic function and are involved in movement preparation as they are needed to focus on the target. Indeed, dopamine enhances the stability of task-relevant representations by promoting distractor resistance [34, 35]. In addition, in patients with PD, reaction time in choice reaction time tasks may improve after levodopa administration [36, 37], although this improvement is not paralleled by a similar improvement of the cognitive network underlying declarative learning [36].

Dopaminergic projections to the frontal cortex are mediated by the mesocortical pathway. In particular orbitofrontal cortex, which seems to guide behavior based on expected reward, not only receives mesocortical dopaminergic inputs, but also may modulate dopaminergic output of the ventral-tegmental area, where most of the mesocortical neurons originate [9]. Recent animal studies underline the importance of dopamine in high level cognitive functioning, working memory, risk-based decision making, and attention [8] but also for the process of attending external stimuli and for associative learning [6]. These evidences underline the existence of a shared functional background between the orbitofrontal cortex and the cognitive processes underlying reaction time as evaluated in our motor task.

Despite encouraging results in animal models, the role of dopamine in human cognition is still poorly understood. Dopaminergic therapy in particular, despite being usually effective on motor symptoms, may produce supranormal dopaminergic transmission in limbic and associative cortical networks, determining a variable interplay between failure and adaptation of different brain circuitry [38]. Since PD patients in this study were all drug naïve, the role of endogenous dopamine has not been affected by dopaminergic drugs. An explanation for the inverse correlation between dopaminergic activity in the lateral orbitofrontal cortex and reaction time could reside in a compensatory supranormal dopaminergic activity mostly in those patients showing a lower reaction time. Conversely, the lack of correlation between reaction time and UPDRS-III, a clinical scale that estimates motor impairment in PD patients, favors the hypothesis that reaction time is not necessarily related to overall motor impairment [11]. Even in naïve PD, the degree of impairment of the dopaminergic system is very variable among patients; similarly the compensatory mesocortical activity is likely to be higher in PD patients with lower levels of endogenous dopamine and vice versa. The absent correlation between dopaminergic activity in the lateral orbitofrontal cortex and reaction in the control group is probably a consequence of the integrity of the dopaminergic system in patients with essential tremor, determining much more uniform dopamine levels and reduced variability among subjects.

The possible role of reward, which is mediated by dopaminergic pathways in the orbitofrontal cortex, has been demonstrated in both human [7, 9] and animal studies [6, 8, 10]. Even if the motor task we used has not been specifically designed to study reward, it was somewhat challenging because of the short time interval between targets and the positive feedback of the target turning gray after a successful hit. Such features could have modulated reaction time in relation to the individual expected reward, determining a shorter (better) reaction time in those PD patients where dopamine levels in the lateral orbitofrontal cortex were higher, probably because of more preserved and upregulated mesocortical projections [9].

We found that reaction time correlated with dopaminergic activity of the left lateral orbitofrontal cortex but not with the right one. Importantly, such correlation was lost if the brain hemispheres were flipped in order to match the more affected side of PD patients. This could mean that the correlation involves the left cortex independently on the body side where the motor features are more evident. Lateralization of dopaminergic activity has already been shown in the striatum of PD patients as measured by SPECT with ^123^I-b-CIT [39] and ^18^F-DOPA [3] while considering frontal cognitive tasks. It is likely that the reaction time correlates that we have studied are actually localized in the left lateral orbitofrontal cortex; however we cannot exclude that such lateralization is related to the use of the right arm while performing the motor task. Having tested the patients only with the right arm is of course a limitation of this study; investigating the motor performance bilaterally may be useful in future studies to better understand motor and cognitive effect of an asymmetric dopaminergic brain activity.

Although ^18^F-DOPA uptake is mainly related to dopaminergic transmission, it is not a specific ligand for dopamine neurons since its uptake is related to the aromatic amino acid decarboxylase (AADC) enzyme, present also in other monoaminergic neurons. It is therefore possible that at least part of the correlation we found could be related also to noradrenergic or serotoninergic activity in the same brain area [4]. Even if PD is mainly a dopamine-related disorder and therefore dopamine is more likely responsible for the present findings, the role of other monoamines is intriguing. Noradrenaline in particular has a role in vigilance and attention and, like dopamine, is reduced in PD brains [40]. On the other hand frontal serotonin is involved in response inhibition, which is modulated by serotonin reuptake inhibitors in PD [41].

In conclusion the present study contributes to the understanding of the neural mechanisms underlying reaction time and the related cognitive processes such as attention, stimulus perception, movement preparation, and the possible role of reward. Our results help to understand the role of left lateral orbitofrontal cortex in patients with Parkinson's disease.

## Figures and Tables

**Table 1:** Patients demographic data.

Patient	Group	Sex	Age	MMSE	UPDRS-III	MAH
1	PD	M	65	29	22	R
2	PD	M	65	28	21	R
3	PD	M	64	30	9	R
4	PD	M	73	29	14	L
5	PD	F	74	29	12	R
6	PD	M	66	29	11	L
7	PD	M	65	30	10	L
8	PD	F	76	29	23	R
9	PD	M	68	27	19	R
10	PD	M	74	29	23	R
11	PD	M	60	28	14	R
12	PD	M	68	28	12	L
13	PD	M	47	30	15	L
14	PD	F	74	30	18	R
15	PD	M	65	30	15	R
16	PD	M	62	28	16	R
1	ET	M	73	30		
2	ET	F	68	27		
3	ET	M	84	29		
4	ET	F	79	29		
5	ET	M	77	29		
6	ET	F	57	29		
7	ET	F	54	30		
8	ET	F	60	29		
9	ET	M	67	30		
10	ET	F	63	29		

MAH: more affected hemisphere.

**Table 2:** Results of correlation analysis between mean reaction time (RT) and dopaminergic activity as assessed through ^18^F-DOPA PET in de novo Parkinson's disease patients.

Analysis	Cluster level			Peak level					
	Cluster extent	Corrected *p* value	Cortical region	Maximum *Z* score	Talairach coordinates			Cortical region	BA
RT	126	0.05	L-frontal	6.02	−42	40	−19	Middle frontal gyrus	11
			L-frontal	5.59	−46	30	−17	Inferior frontal gyrus	47
			L-temporal	4.83	−48	20	−20	Superior temporal gyrus	38

Uncorrected *p* < 0.001 at peak level and *p* < 0.05, false discovery rate corrected at cluster level, were accepted as statistically significant. In the “cluster level” section on the left, for each cluster found to be statistically significant are reported the number of voxels, the corrected *p* value, and the cortical region where the cluster is found. In the “peak level” section on the right, for each significant cluster are reported the peak coordinates and *Z* score, the corresponding cortical region and Brodmann area (BA).

## References

[1] Botha H., Carr J. (2012). Attention and visual dysfunction in Parkinson's disease. *Parkinsonism and Related Disorders*.

[2] Gaenslen A., Wurster I., Brockmann K. (2014). Prodromal features for Parkinson's disease—baseline data from the TREND study. *European Journal of Neurology*.

[3] Cheesman A. L., Barker R. A., Lewis S. J. G., Robbins T. W., Owen A. M., Brooks D. J. (2005). Lateralisation of striatal function: evidence from 18F-dopa PET in Parkinson's disease. *Journal of Neurology, Neurosurgery and Psychiatry*.

[4] Moore R. Y., Whone A. L., Brooks D. J. (2008). Extrastriatal monoamine neuron function in Parkinson's disease: an 18F-dopa PET study. *Neurobiology of Disease*.

[5] Sawamoto N., Piccini P., Hotton G., Pavese N., Thielemans K., Brooks D. J. (2008). Cognitive deficits and striato-frontal dopamine release in Parkinson's disease. *Brain: A Journal of Neurology*.

[6] Garske A. K., Lawyer C. R., Peterson B. M., Illig K. R. (2013). Adolescent changes in dopamine D1 receptor expression in orbitofrontal cortex and piriform cortex accompany an associative learning deficit. *PLoS ONE*.

[7] Joutsa J., Martikainen K., Niemelä S. (2012). Increased medial orbitofrontal [^18^F]fluorodopa uptake in Parkinsonian impulse control disorders. *Movement Disorders*.

[8] Parker K. L., Alberico S. L., Miller A. D., Narayanan N. S. (2013). Prefrontal D1 dopamine signaling is necessary for temporal expectation during reaction time performance. *Neuroscience*.

[9] Takahashi Y. K., Roesch M. R., Wilson R. C. (2011). Expectancy-related changes in firing of dopamine neurons depend on orbitofrontal cortex. *Nature Neuroscience*.

[10] Winstanley C. A., Zeeb F. D., Bedard A. (2010). Dopaminergic modulation of the orbitofrontal cortex affects attention, motivation and impulsive responding in rats performing the five-choice serial reaction time task. *Behavioural Brain Research*.

[11] Marinelli L., Perfetti B., Moisello C. (2010). Increased reaction time predicts visual learning deficits in Parkinson's disease. *Movement Disorders*.

[12] Brooks D. J., Playford E. D., Ibanez V. (1992). Isolated tremor and disruption of the nigrostriatal dopaminergic system: an ^18^F-dopa PET study. *Neurology*.

[13] Marinelli L., Pelosin E., Trompetto C. (2011). In idiopathic cervical dystonia movement direction is inaccurate when reaching in unusual workspaces. *Parkinsonism and Related Disorders*.

[14] Luxen A., Perlmutter M., Bida G. T. (1990). Remote, semiautomated production of 6-[18F]fluoro-L-dopa for human studies with PET. *Applied Radiation and Isotopes*.

[15] Dhawan V., Ma Y., Pillai V. (2002). Comparative analysis of striatal FDOPA uptake in Parkinson's disease: ratio method versus graphical approach. *Journal of Nuclear Medicine*.

[16] Kumakura Y., Cumming P. (2009). PET studies of cerebral levodopa metabolism: a review of clinical findings and modeling approaches. *The Neuroscientist*.

[17] Ishikawa T., Dhawan V., Chaly T. (1996). Clinical significance of striatal DOPA decarboxylase activity in Parkinson's disease. *Journal of Nuclear Medicine*.

[18] Jokinen P., Helenius H., Rauhala E., Brück A., Eskola O., Rinne J. O. (2009). Simple ratio analysis of ^18^F-fluorodopa uptake in striatal subregions separates patients with early parkinson disease from healthy controls. *Journal of Nuclear Medicine*.

[19] Jaimini A., Tripathi M., D'Souza M. M. (2013). Utility of intrastriatal ratios of FDOPA to differentiate idiopathic Parkinson's disease from atypical parkinsonian disorders. *Nuclear Medicine Communications*.

[20] Eshuis S. A., Jager P. L., Maguire R. P., Jonkman S., Dierckx R. A., Leenders K. L. (2009). Direct comparison of FP-CIT SPECT and F-DOPA PET in patients with Parkinson's disease and healthy controls. *European Journal of Nuclear Medicine and Molecular Imaging*.

[21] Eshuis S. A., Maguire R. P., Leenders K. L., Jonkman S., Jager P. L. (2006). Comparison of FP-CIT SPECT with F-DOPA PET in patients with de novo and advanced Parkinson's disease. *European Journal of Nuclear Medicine and Molecular Imaging*.

[22] Wahlund L. O., Barkhof F., Fazekas F. (2001). A new rating scale for age-related white matter changes applicable to MRI and CT. *Stroke*.

[23] Gispert J. D., Pascau J., Reig S. (2003). Influence of the normalization template on the outcome of statistical parametric mapping of PET scans. *NeuroImage*.

[24] Morbelli S., Piccardo A., Villavecchia G. (2010). Mapping brain morphological and functional conversion patterns in amnestic MCI: a voxel-based MRI and FDG-PET study. *European Journal of Nuclear Medicine and Molecular Imaging*.

[25] Weeks R. A., Cunningham V. J., Piccini P., Waters S., Harding A. E., Brooks D. J. (1997). 11C-diprenorphine binding in Huntington's disease: a comparison of region of interest analysis with statistical parametric mapping. *Journal of Cerebral Blood Flow and Metabolism*.

[26] Bouilleret V., Semah F., Biraben A. (2005). Involvement of the basal ganglia in refractory epilepsy: an 18F-fluoro-L-DOPA PET study using 2 methods of analysis. *Journal of Nuclear Medicine*.

[27] http://marsbar.sourceforge.net/.

[28] Oishi N., Udaka F., Kameyama M., Sawamoto N., Hashikawa K., Fukuyama H. (2005). Regional cerebral blood flow in Parkinson disease with nonpsychotic visual hallucinations. *Neurology*.

[29] brainmap.org http://brainmap.org/.

[30] Lancaster J. L., Woldorff M. G., Parsons L. M. (2000). Automated Talairach Atlas labels for functional brain mapping. *Human Brain Mapping*.

[31] Berardelli A., Rothwell J. C., Thompson P. D., Hallett M. (2001). Pathophysiology of bradykinesia in Parkinson's disease. *Brain*.

[32] Montgomery E. B., Baker K. B., Lyons K., Koller W. C. (2000). Motor initiation and execution in essential tremor and Parkinson's disease. *Movement Disorders*.

[33] Dujardin K., Tard C., Duhamel A. (2013). The pattern of attentional deficits in Parkinson's disease. *Parkinsonism and Related Disorders*.

[34] Cools R., Miyakawa A., Sheridan M., D'Esposito M. (2010). Enhanced frontal function in Parkinson's disease. *Brain*.

[35] Durstewitz D., Seamans J. K. (2008). The dual-state theory of prefrontal cortex dopamine function with relevance to catechol-*O*-methyltransferase genotypes and schizophrenia. *Biological Psychiatry*.

[36] Ghilardi M. F., Feigin A. S., Battaglia F. (2007). L-Dopa infusion does not improve explicit sequence learning in Parkinson's disease. *Parkinsonism and Related Disorders*.

[37] Pullman S. L., Watts R. L., Juncos J. L., Chase T. N., Sanes J. N. (1988). Dopaminergic effects on simple and choice reaction time performance in Parkinson's disease. *Neurology*.

[38] Redgrave P., Vautrelle N., Stafford T. (2014). Interpretive conundrums when practice doesn't always make perfect. *Movement Disorders*.

[39] Duchesne N., Soucy J.-P., Masson H., Chouinard S., Bédard M.-A. (2002). Cognitive deficits and striatal dopaminergic denervation in Parkinson's disease: a single photon emission computed tomography study using 123Iodine-*β*-CIT in patients ON and OFF levodopa. *Clinical Neuropharmacology*.

[40] Stern Y., Mayeux R., Côté L. (1984). Reaction time and vigilance in Parkinson's disease. Possible role of altered norepinephrine metabolism. *Archives of Neurology*.

[41] Ye Z., Altena E., Nombela C. (2014). Selective serotonin reuptake inhibition modulates response inhibition in Parkinson's disease. *Brain*.

